# Autophagy Adaptor Protein p62/SQSTM1 and Autophagy-Related Gene Atg5 Mediate Autophagosome Formation in Response to *Mycobacterium tuberculosis* Infection in Dendritic Cells

**DOI:** 10.1371/journal.pone.0086017

**Published:** 2013-12-23

**Authors:** Shintaro Seto, Kunio Tsujimura, Toshinobu Horii, Yukio Koide

**Affiliations:** 1 Department of Infectious Diseases, Hamamatsu University School of Medicine, Hamamatsu, Shizuoka, Japan; University of Maryland, United States of America

## Abstract

*Mycobacterium tuberculosis* is an intracellular pathogen that can survive within phagocytic cells by inhibiting phagolysosome biogenesis. However, host cells can control the intracellular *M. tuberculosis* burden by the induction of autophagy. The mechanism of autophagosome formation to *M. tuberculosis* has been well studied in macrophages, but remains unclear in dendritic cells. We therefore characterized autophagosome formation in response to *M. tuberculosis* infection in dendritic cells. Autophagy marker protein LC3, autophagy adaptor protein p62/SQSTM1 (p62) and ubiquitin co-localized to *M. tuberculosis* in dendritic cells. Mycobacterial autophagosomes fused with lysosomes during infection, and major histcompatibility complex class II molecules (MHC II) also localized to mycobacterial autophagosomes. The proteins p62 and Atg5 function in the initiation and progression of autophagosome formation to *M. tuberculosis*, respectively; p62 mediates ubiquitination of *M. tuberculosis* and Atg5 is involved in the trafficking of degradative vesicles and MHC II to mycobacterial autophagosomes. These results imply that the autophagosome formation to *M. tuberculosis* in dendritic cells promotes the antigen presentation of mycobacterial peptides to CD4^+^ T lymphocytes via MHC II.

## Introduction

*Mycobacterium tuberculosis* is the causative agent of tuberculosis and infects one-third of the world’s population. *M. tuberculosis* is an intracellular bacterium that can survive within infected the phagocytic cells and its ability to block phagolysosome biogenesis [1] may contribute to its persistence within host cells. A number of reports have demonstrated that mycobacteria inhibit phagolysosome biogenesis by arresting phagosome maturation [2]. Our studies have supported these findings by showing that *M. tuberculosis* infection modulates the trafficking of Rab GTPases that regulate phagosome maturation, thus resulting in the inhibition of phagolysosome biogenesis in macrophages [3-6].

Despite the intracellular proliferation of *M. tuberculosis* in macrophages, the interaction of infected macrophages and T lymphocytes promotes the elimination of *M. tuberculosis* [7,8]. This process illustrates the close interaction between the innate and adaptive immunity systems in pathogens clearance. For example, macrophages and dendritic cells (DC) function as professional antigen-presenting cells in adaptive immunity, and presentation of mycobacterial antigens to T lymphocytes by major histocompatibility complex (MHC) molecules on DC induces acquired immune responses. Consequently, interferon (IFN)-γ secreted by CD4^+^ T lymphocytes induces granuloma formation, which restricts and controls the burden of infecting bacilli [9,10]. It is widely accepted that T lymphocytes are activated by DC that ingest material containing mycobacterial antigens, including apoptotic cells [11,12] and exosomes [13]. However, the contribution of directly infected DC in the activation of T lymphocytes remains unclear.

Autophagy is a unique lysosomal degradation pathway for the destruction of cytoplasmic materials. This pathway is also triggered by invasion of intracellular pathogen and contributes to the protection of host cells [14]. Autophagy also controls the proliferation of *M. tuberculosis* in macrophages following its infection [15]. Autophagy induced by exogenous stimulations, such as starvation, rapamycin, vitamin D3 and IFN-γ, can eliminate the infecting mycobacteria in macrophages [16-18]. In DC, the activation of autophagy also contributes to the presentation of mycobacterial antigen [19,20].

Macrophages and DC respond differentially to *M. tuberculosis* infection; Tailleux et al. reported that the proliferation of *M. tuberculosis* is restricted in DC, but not in macrophages [21]. The authors also demonstrated that *M. tuberculosis*-infected DC can present mycobacterial antigens to CD4^+^ T lymphocytes, but mycobacterial phagosomes neither undergo acidification nor fuse with lysosomes [21], suggesting a unique membrane trafficking of mycobacterial phagosomes in infected DC. Since autophagy is involved in antigen presentation via MHC II [22], autophagy may promote the presentation of mycobacterial antigens in DC. However, it is unclear whether selective autophagy actually occurs in response to mycobacteria infection in DC. In this study, we demonstrated that *M. tuberculosis* infection induces selective autophagy in DC and that mycobacterial autophagosomes fuse with lysosomes and recruit MHC II. These results suggest that selective autophagosome formation targets to the *M. tuberculosis* bacilli in infected DC, and this is then followed by autolysosome biogenesis.

## Materials and Methods

### Ethics statement

Animal experiments in this study were approved by the Hamamatsu University School of Medicine Animal Care Committees at the Center Animal Care facility (permit number: 2012074). Mice were sacrificed by cervical dislocation and all efforts were made to minimize suffering.

### Cell and bacterial cultures

Murine bone marrow-derived macrophages (BMM) or DC (BMDC) were differentiated from bone marrow cells of C57BL/6 mice by culturing in DMEM supplemented with 10% L929-conditional medium and 10% fetal bovine serum (FBS), 25 μg/ml penicillin G and 25 μg/ml streptomycin or RPMI 1640 supplemented with 10% FBS, 20 ng/ml granulocyte macrophage-colony stimulating factor (GM-CSF, PeproTech, Rocky Hill, NJ) and antibiotics, respectively [23,24]. At day 7, cultured BMM or BMDC were 90% CD11b-positive or 80% CD11c-positive, respectively. DC2.4 cells [25] were kindly provided by Dr. Kenneth Rock (University of Massachusetts Medical Center, Worcester, MA) and maintained in RPMI 1640 supplemented with 10% FBS and antibiotics. JAWSII cells were obtained from the American Type Culture Collection and maintained in RPMI 1640 supplemented with 10% FBS, 5 ng/ml GM-CSF and antibiotics. *M. tuberculosis* Erdman and *Mycobacterium bovis* strain Bacillus Calmette-Guérin (BCG) Tokyo were obtained from the Japan Research Institute of Tuberculosis (Tokyo, Japan) and Japan BCG Laboratory (Tokyo, Japan), respectively. Mycobacteria were grown to mid-logarithmic phase in 7H9 medium supplemented with 10% Middlebrook ADC (BD Biosciences, San Jose, CA), 0.5% glycerol, and 0.05% Tween-80 (*Mycobacterium* complete medium) at 37°C. Mycobacteria transformed with a plasmid encoding DsRed were grown in *Mycobacterium* complete medium containing 25 μg/ml kanamycin. To label *M. tuberculosis* with Alexa Fluor 405, mycobacteria were incubated with Alexa Fluor 405 succinimidyl ester (Invitrogen, Carlsbad, CA) as described previously [5].

### RNA interference

siRNA duplexes were synthesized by Sigma-Aldrich (St. Louis, MO) using the following templates: p62#1, sense 5’-GCAUUGAGGUUGACAUUGATT-3’, antisense 5’-UCAAUGUCAACCUCAAUGCTT-3’; p62#2, sense 5’-CAUCUUCCGCAUCUACAUUTT-3’, antisense 5’-AAUGUAGAUGCGGAAGAUGTT-3’; Atg16L#1, sense 5’-GAAUUACAAGCAUUGAUUGAAUUTT-3’, antisense 5’-AAUUCAAUGCUUGUAAUUCTT-3’ ’; Atg16L#2, sense 5’-CCUAUUAGCAGCUUCAAAUTT-3’, antisense 5’-AUUUGAAGCUUGCUAAUAGGTT-3’. siRNA for Atg5 was also synthesized as previously described [26]. Mission siRNA universal negative control (Sigma-Aldrich) was used as the control. Transfection of DC with siRNA duplexes were performed using Lipofectamine RNAiMAX (Invitrogen) according to the manufacturer’s instructions.

### Antibodies

Mouse anti-LC3 monoclonal antibody (４E12, MBL, Nagoya, Japan), rabbit anti-LC3 polyclonal antibody (L7543, Sigma-Aldrich), rabbit anti-p62 polyclonal antibody (PM045, MBL), mouse anti-ubiquitin monoclonal antibody (FK2, MBL), mouse anti-tubulin monoclonal antibody (DM1A, Sigma-Aldrich), rabbit anti-Atg5 polyclonal antibody (A0856, Sigma-Aldrich), mouse anti-Rab7 monoclonal antibody (Rab7-117, Abcam, Cambridge, United Kingdom), mouse anti-actin monoclonal antibody (AC-15, Sigma-Aldrich), rat anti-mouse LAMP1 monoclonal antibody (1D4B, SouthernBiotech, Birmingham, AL), rat anti-mouse MHC II (I-A/I-E) monoclonal antibody (M5/114.15.2, eBioscience, San Diego, CA) and rabbit anti-Atg16L polyclonal antibody (PM040, MBL) were used as primary antibodies. Alexa Fluor 488- or Alexa Fluor 546-conjugated anti-IgG antibodies (Invitrogen) and horseradish peroxidase-conjugated anti-IgG antibodies (Dako, Glostrup, Denmark) were used as secondary antibodies.

### Fluorescence microscopy and immunoblotting analysis

Immunofluorescence microscopic analysis was performed as previously described [3]. DC were stained with anti-LC3 monoclonal antibody (1:25 v/v), anti-p62 antibody (1:25 v/v), anti-ubiquitin antibody (1:25 v/v), anti-LAMP1 antibody (1:10 v/v) or anti-MHC II antibody (1:25 v/v). For labeling degradative vesicles with DQ-BSA green (Invitrogen), infected DC were incubated with the fluorescent dye at 10 μg/ml for 6 h before fixation. Fluorescence microscopy was performed using an LS-1 laser scanning confocal microscope (LSCM; Yokogawa, Tokyo, Japan). Images were processed by ImageJ (http://rsbweb.nih.gov/ij/) to verify co-localization between mycobacteria and autophagic marker proteins (Figure S1). For immunoblot analysis, DC lysates were extracted using cell lysis buffer containing 25 mM Tris-HCl pH 7.6, 150 mM NaCl, 1% NP-40, 1% sodium deoxycholate, 0.1% SDS, 100 μM vanadate, and protease inhibitor cocktail (Roche, Mannheim, Germany). Cell lysates were separated by SDS-polyacrylamide gel electrophoresis (SDS-PAGE) and then subjected to immunoblot analysis using anti-LC3 polyclonal antibody (1:500 v/v), anti-tubulin antibody (1:1000 v/v), anti-p62 antibody (1:500), anti-Atg5 antibody (1:300 v/v), anti-Rab7 antibody (1:300 v/v), anti-actin antibody (1:1000 v/v) or anti-Atg16L antibody (1:300 v/v). Band intensities from three independent experiments were quantified using ImageJ.

### Infection with mycobacteria

For observation by fluorescence microscopy, siRNA-transfected DC were scraped 48 h after transfection and grown on round coverslips in 12-well plates for a further 12 h. Mycobacteria were washed three times with PBS containing 0.05% Tween-80 and suspended in DMEM containing 10% FBS at a multiplicity of infection (MOI) of 30. Aliquots of 1 ml of bacterial suspension were applied to 2 × 10^5^ DC on coverslips in 12-well plates, followed by centrifugation at 150 × *g* for 5 min and incubation for 10 min at 37°C. Infected cells on coverslips were washed three times with RPMI1640 to remove non-infecting bacteria and then incubated with RPMI1640 containing 10% FBS. At the indicated time points, infected cells were fixed with 3% paraformaldehyde in PBS. We confirmed that the viability of non-infected or infected DC was more than 90% at 24 h postinfection (p.i.) using a LIVE/DEAD cell viability kit (Invitrogen) by fluorescence microscopy. For immunoblot analysis to detect LC3 processing, DC2.4 cells in 6-well plates were infected with *M. tuberculosis* or BCG at an MOI of 10 and incubated for 4 h at 37°C. Infected cells were washed with RPMI 1640 to remove non-infected bacteria and then incubated with RPMI 1640 containing 10% FBS for a further 20 h. Infected cells were washed three times with PBS and lysed using a cell lysis buffer.

### Thin-section electron microscopy

DC2.4 cells transfected with siRNA in 6-well plates were infected with *M. tuberculosis* for 24 h, and then fixed with 1% glutaraldehyde in 0.2 M cacodylic acid buffer. Fixed DC were incubated with 0.1% (w/v) osmium tetroxide. Dehydration was performed by a series of ethanol washes, followed by treatment with propylene oxide. Samples were embedded in Qetol812 resin (Oken, Tokyo, Japan) according to the manufacturer's protocol. Thin sections were cut with diamond knives and mounted on copper grids. Samples on grids were counter stained with 2% (w/v) uranyl acetate and then observed with a JEM-1220 electron microscope (JEOL, Tokyo, Japan).

### Statistical analysis

Paired or unpaired two-tailed Student’s *t*-test was applied to assess the statistical significance of differences between the two groups. To assess the proportions of fluorescence-positive mycobacteria by fluorescence microscopy or those of mycobacteria in multi-membrane structures by thin-section electron microscopy, three independent experiments were conducted, and more than 200 or 50 phagosomes were counted for each condition, respectively.

## Results

### Autophagosome formation to *M. tuberculosis* in DC

**Figure 1:**
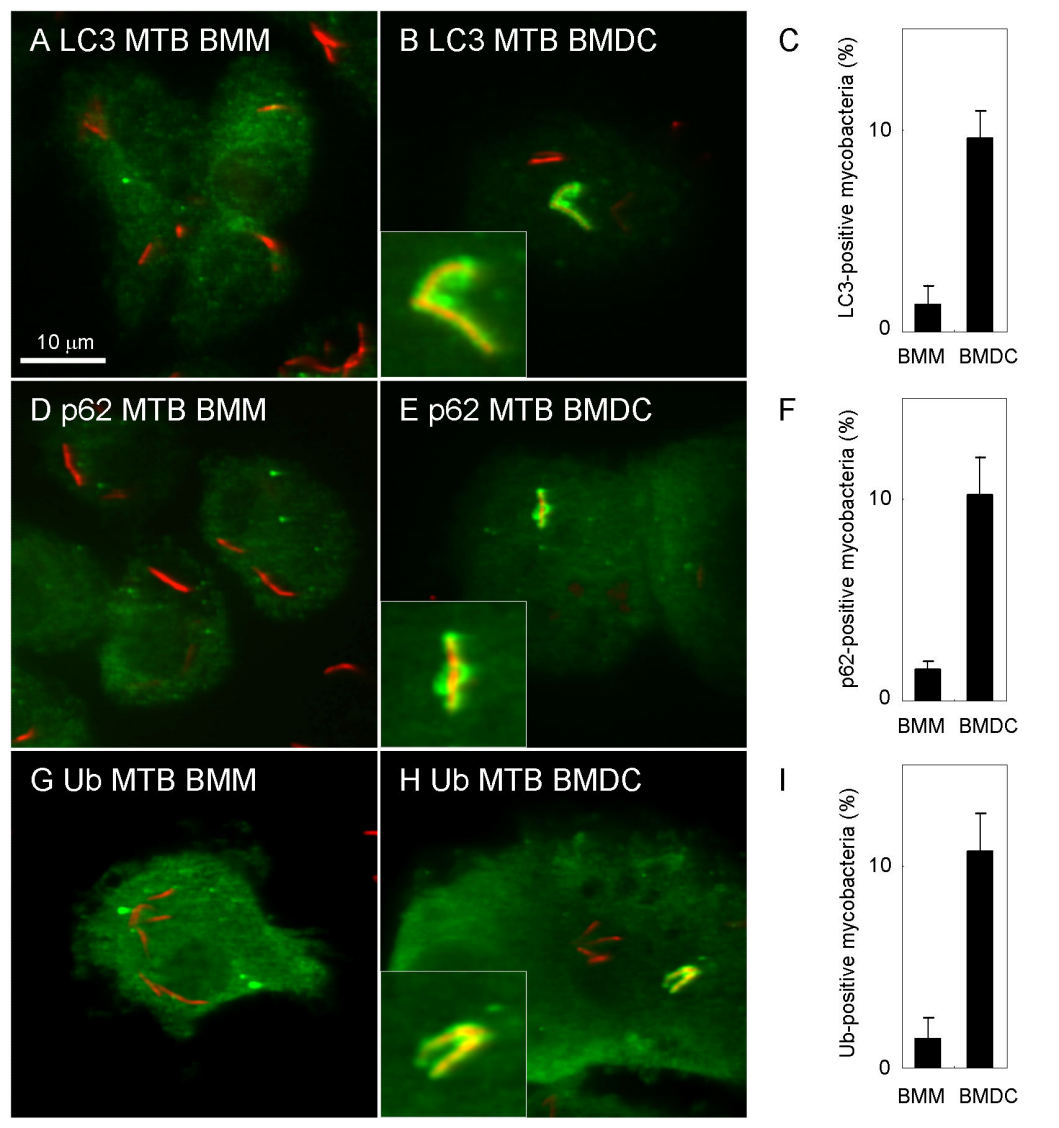
Localization of autophagosome markers to *M. tuberculosis* in BMDC. Bone marrow-derived macrophages (BMM) or dendritic cells (BMDC) were infected with DsRed-expressing *M. tuberculosis* for 24 h and immunostained with anti-LC3 (A, B), anti-p62 (D, E) or anti-ubiquitin (G, H) antibody. The proportion of LC3-positive (C), p62-positive (F) or ubiquitin-positive (I) *M. tuberculosis* in BMM or BMDC is also shown. Data represent the mean and SD of three independent experiments.

To assess whether autophagosomes are formed around *M. tuberculosis* bacilli in macrophages and DC, we infected BMM or BMDC with *M. tuberculosis* and by immunofluorescence microscopy, we examined the localization of the autophagic marker protein, LC3 [27], around *M. tuberculosis* (Figure 1A–C). Endogenous LC3 localized to approximately 10% of infecting mycobacteria in BMDC at 24 h p.i. In contrast, LC3 did not localize to mycobacteria in BMM at 24 h p.i. as previously described [26,28]. We further examined the localization of an autophagy adaptor protein, p62/SQSTM1 (p62) and ubiquitin to mycobacteria in BMM or BMDC (Figure 1D–I). p62 is involved in targeting ubiquitinated intracellular bacteria to the selective autophagic pathway [29] and is responsible for autophagic elimination of mycobacteria in macrophages [30]. Results showing that LC3, p62 and ubiquitin were recruited to mycobacteria in BMDC but not in BMM at 24 h p.i. These results suggest that autophagy markers targets *M. tuberculosis* in BMDC but not BMM. Next, the recruitment of LC3, p62 or ubiquitin to BCG in BMDC was examined (data not shown). The BCG genome lacks several gene clusters, termed regions of difference (RD) [31] including the RD-1 region that contains genes encoding the secretion machinery of early secretory antigenic target (ESAT)-6 and other secretion proteins (ESX-1) [32]. Infection with BCG did not induce the recruitment of LC3, p62 and ubiquitin to mycobacteria in BMDC, suggesting that BCG does not induce the recruitment of autophagy markers in BMDC.

**Figure 2:**
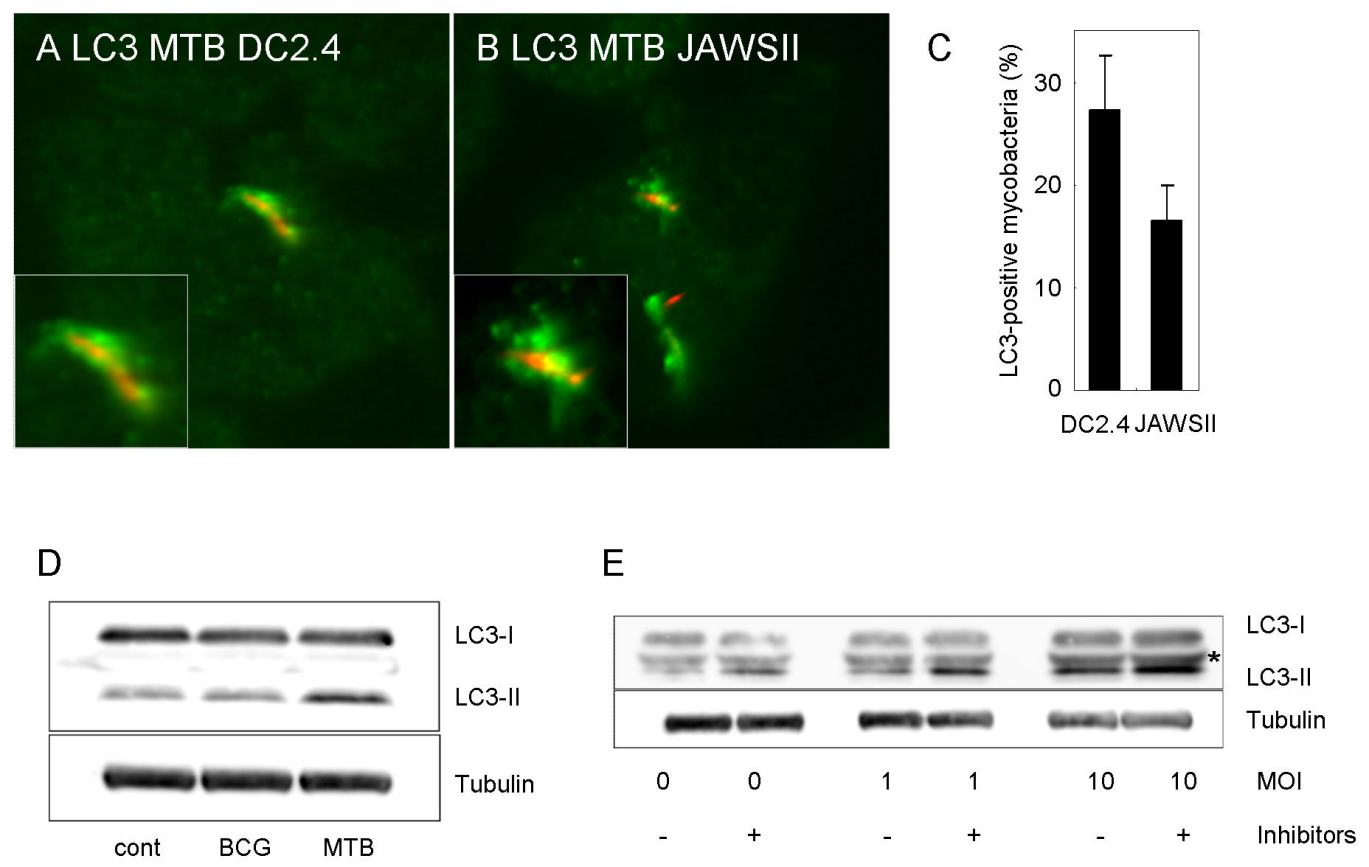
Autophagy induction in response to infection of *M. tuberculosis* in DC cell lines. (A, B, C) LC3 localization to mycobacteria in dendritic cell line. DC2.4 (A) or JAWSII (B) cells were infected with *M. tuberculosis* for 24 h and immunostained with anti-LC3 antibody. The proportion of LC3-positive mycobacteria in these cell lines is also shown (C). Data represent the mean and SD of three independent experiments. (D) Immunoblot analysis of LC3 processing in DC2.4 cells infected with *M. tuberculosis* or *M. bovis* BCG for 24 h. (E) Autophagic flux in *M. tuberculosis*-infected DC2.4 cells. DC2.4 cells treated with or without protease inhibitors, E64d (10 μg/ml) and pepstatin A (10 μg/ml), were infected with *M. tuberculosis* for 24 h at the indicated multiplicity of infection (MOI). *Non-specific band.

To analyze autophagosome formation to *M. tuberculosis* in DC, we infected two available DC lines, DC2.4 [25] and JAWSII with mycobacterial bacilli and immunostained with anti-LC3 antibody (Figure 2A-C). We found the proportion of LC3-positive mycobacteria in these cell lines to be greater than that in BMDC at 24 h p.i., suggesting that these DC lines are more susceptible to mycobacterial infection in autophagsome formation. Additionally, we found that p62 and ubiquitin also localized to mycobacteria in these DC lines (data not shown). We then examined the processing of LC3 in BCG- or *M. tuberculosis*-infected DC2.4 cells by immunoblot analysis (Figure 2D). Infection with *M. tuberculosis* increased the processing of LC3 in DC2.4, whereas there was no significant increase in LC3 processing upon BCG infection. To evaluate the lysosome-dependent autophagic degradation (autophagic flux) in *M. tuberculosis* infected DC2.4 cells, we treated these cells with protease inhibitors, E64d and pepstatin A and examined the LC3 processing (Figure 2E). Quantitative analysis revealed that the treatment with protease inhibitors significantly augmented LC3 processing in *M. tuberculosis*-infected DC2.4 cells as well as in non-infected cells (Figure S2), suggesting that *M. tuberculosis* infection did not affect the autophagic flux in DC2.4 cells. Taken together, these results suggest that autophagy is induced in response to *M. tuberculosis* infection, forming autophagosomes around the bacilli in DC2.4 cells.

### Maturation of mycobacterial autophagosomes

**Figure 3:**
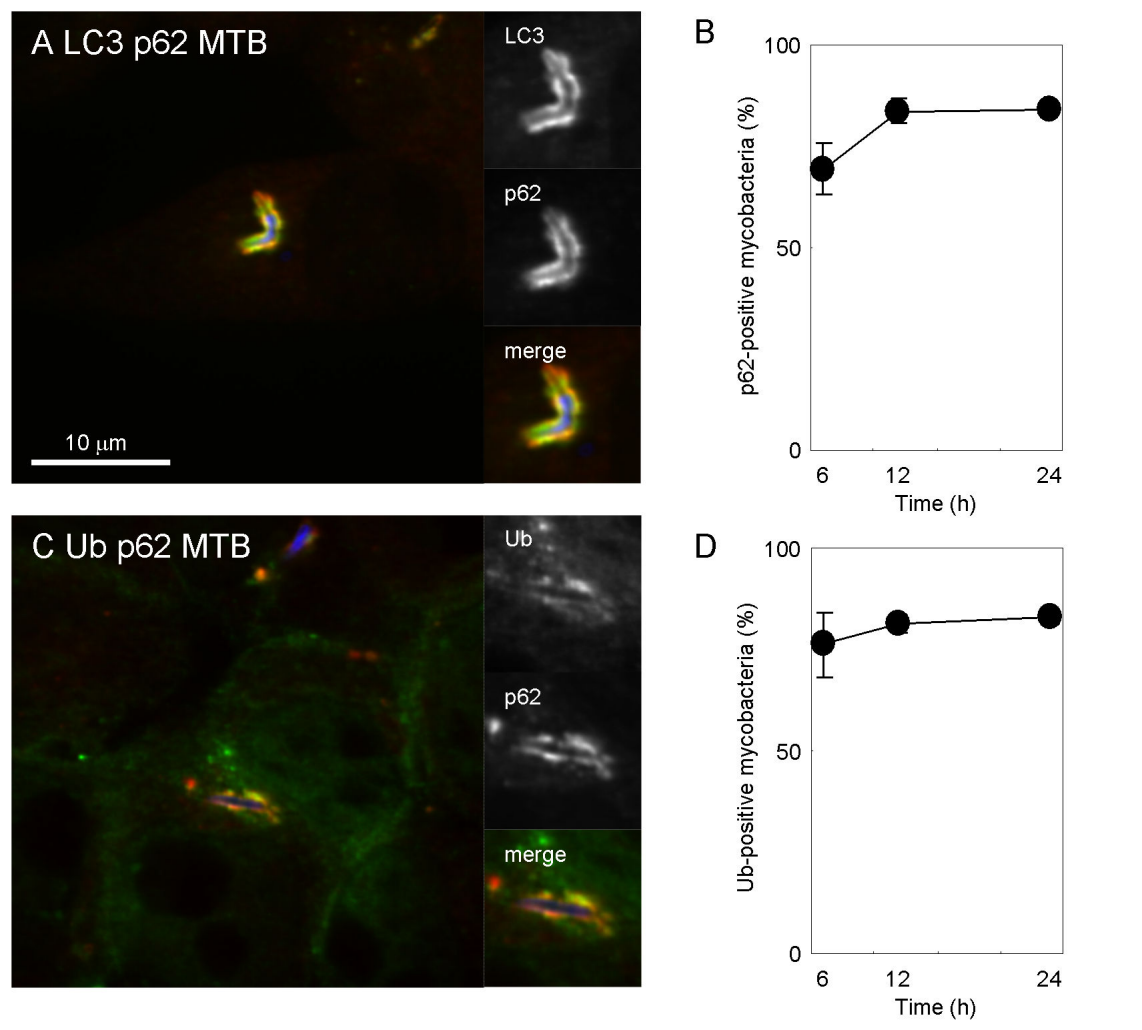
Maturation of mycobacterial autophagosomes in DC2.4 cells. (A) Recruitment of p62 to LC3-positive mycobacteria. DC2.4 cells were infected with Alexa Fluor 405-labeled *M. tuberculosis* (blue) for 24 h and immunostained with anti-LC3 (green) and anti-p62 antibodies (red). (B) The proportion of p62 localization to LC3-positive mycobacteria in DC2.4 cells. (C) Recruitment of ubiquitin to p62-positive mycobacteria. DC2.4 cells were infected with Alexa Fluor 405-labeled *M. tuberculosis* (blue) for 24 h and immunostained with anti-p62 (red) and anti-ubiquitin antibodies (green). (D) The proportion of ubiquitin localization to p62-positive mycobacteria in DC2.4 cells. Data represent the mean and SD of three independent experiments.

We examined the kinetics of localization of LC3, p62 or ubiquitin to *M. tuberculosis* in DC2.4 cells. These autophagic markers localized to only a small population of infected mycobacteria at 2 h p.i., but the numbers of mycobacteria labeled with these markers increased at 6 h p.i (Figure S3). To evaluate the maturation process of mycobacterial autophagosomes in DC, we examined the co-localization of LC3 and p62 to *M. tuberculosis* in DC2.4 cells. Immunofluorescence microscopic analyses revealed that greater than 70% of LC3-positive mycobacteria were also labeled with p62 at 6 h p.i. (Figure 3A, B), and a similar proportion of p62-positive mycobacteria were labeled with ubiquitin (Figure 3C, D). These results suggest that LC3, p62 and ubiquitin co-localize to *M. tuberculosis* in DC.

**Figure 4:**
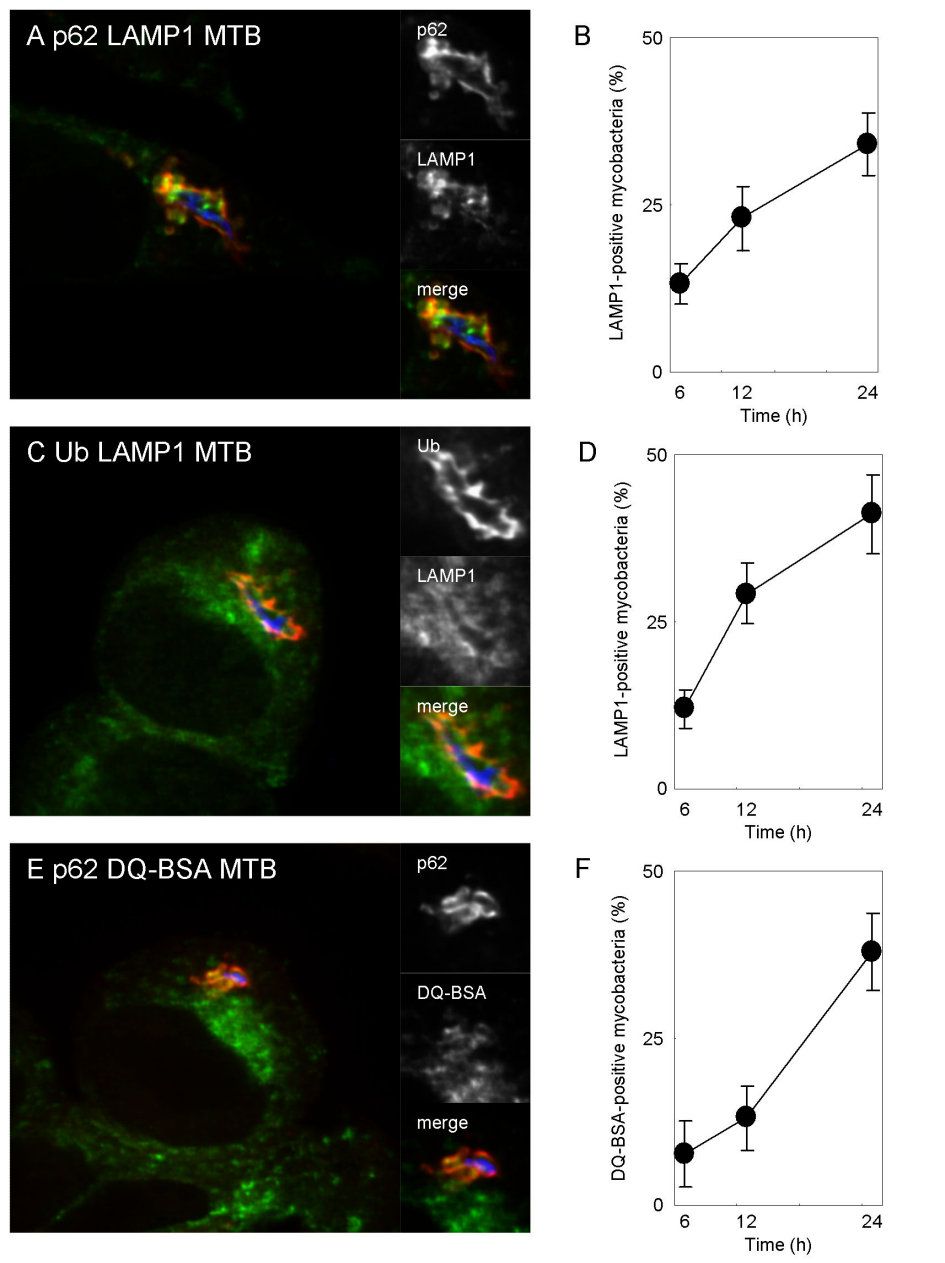
Mycobacterial autolysosome biogenesis. (A, C) Localization of LAMP1 to p62-positive of ubiquitin-positive mycobacteria in DC2.4 cells. DC2.4 cells were infected with Alexa Fluor 405-labeled *M. tuberculosis* (blue) for 24 h and immunostained with anti-LAMP1 (red) and anti-p62 antibodies (green) (A) or anti-LAMP1 (red) and anti-ubiquitin antibodies (green) (C). (B, D) The proportion of LAMP1-localized p62-positive (B) or ubiquitin-positive (D) mycobacteria. (E) Localization of DQ-BSA to mycobacterial autophagosomes. DC2.4 cells were preloaded with DQ-BSA to label degradative vesicles. DC were infected with Alexa Fluor 405-labeled mycobacteria for 24 h and immunostained with anti-p62 antibody. (F) The proportion of DQ-BSA-labeled p62-positive mycobacteria in DC2.4 cells. Data represent the mean and SD of three independent experiments.

To assess the occurrence of autolysosome biogenesis, we examined the co-localization of the lysosomal marker protein LAMP1 to p62-positive or ubiquitin-positive mycobacteria in DC2.4 cells. The localization kinetics of LAMP1 to p62-positive or ubiquitin-positive mycobacteria were slower than that for p62 to LC3-postive mycobacteria or ubiquitin to p62-positive mycobacteria, but the proportion of LAMP1-positive mycobacteria labeled with p62 or ubiquitin increased up to 24 h p.i. (Figure 4A–D). To confirm that mycobacterial autophagosomes fuse with degradative vesicles, we examined the localization of DQ-BSA to mycobacterial autophagosomes (Figure 4E, F and data not shown). In agreement with the results for LAMP1 staining, the proportion of DQ-BSA-positive mycobacteria labeled with p62 or ubiquitin increased up to 24 h p.i. These results suggest that mycobacterial autophagosomes gradually fuses with lysosomes during infection in DC.

### MHC II localizes to mycobacterial autophagosomes

**Figure 5:**
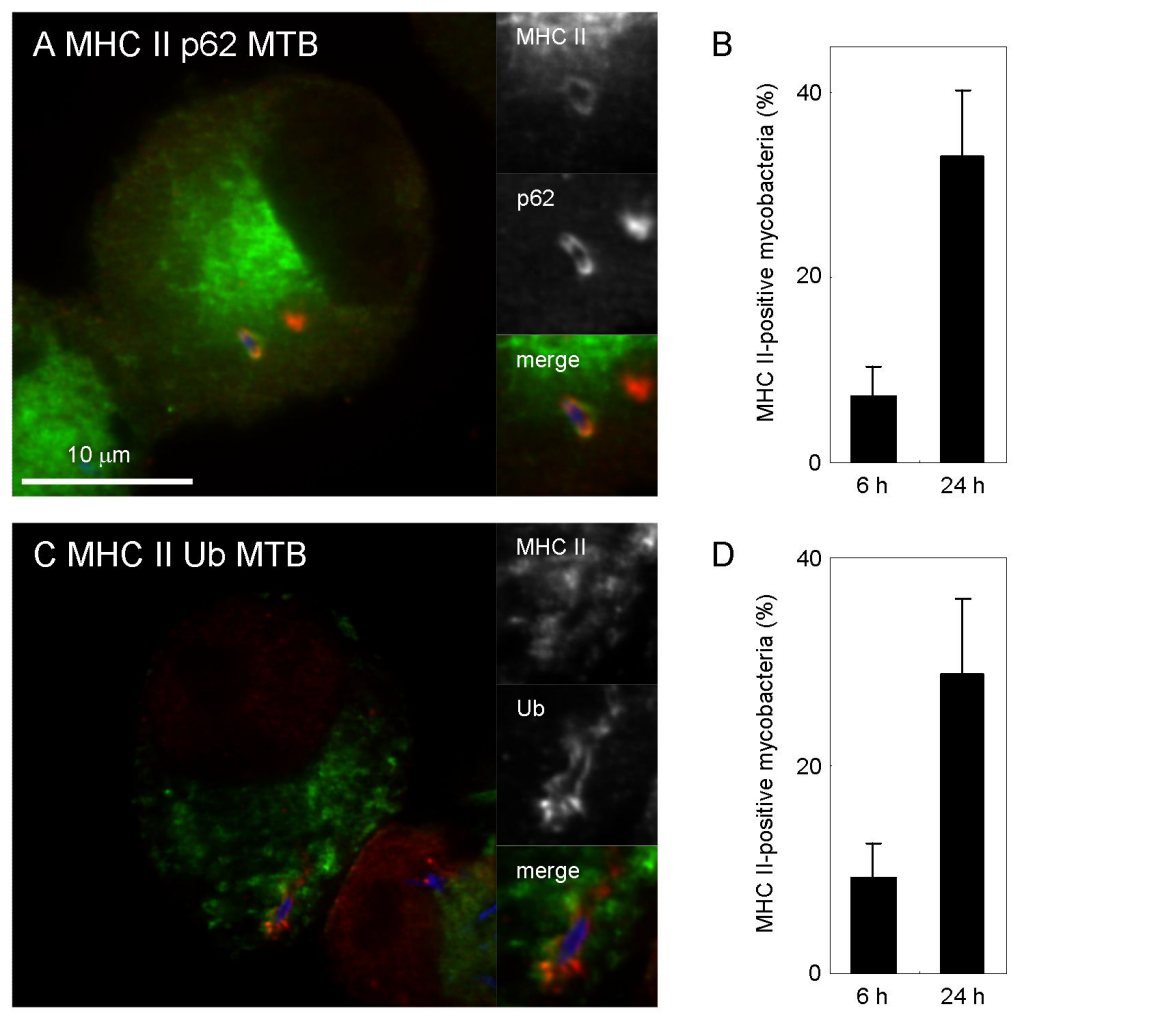
Localization of MHC class II to mycobacterial autophagosomes in DC. (A, C) Localization of MHC class II to p62-positive or ubiquitin-positive mycobacteria in JAWSII cells. JAWSII cells were infected with Alexa Fluor 405-labeled *M. tuberculosis* (blue) for 24 h and immunostained with anti-MHC class II (green) and anti-p62 antibodies (red) (A) or anti-MHC class II (green) and anti-ubiquitin antibodies (red) (C). (B, D) The proportion of MHCII-localized p62-positive (B) orubiquitin-positive (D) mycobacteria in JAWSII cells. Data represent the mean and SD of three independent experiments.

Due to the fact that MHC II localizes to late endosomal and lysosomal compartments [33] and also localizes to autophagosomes [34], we next examined the localization of MHC II to mycobacterial autophagosomes in DC. MHC II localized to p62-positive or ubiquitin-positive *M. tuberculosis* in JAWSII cells (Figure 5A, C). The proportion of MHC II-positive mycobacteria simultaneously labeled with p62 or ubiquitin increased during infection (Figure 5B, D). These results suggest that autophagosome formation stimulates the localization of MHC II to mycobacterial autophagosomes in DC.

### Ubiquitination of mycobacteria is p62-dependent

**Figure 6:**
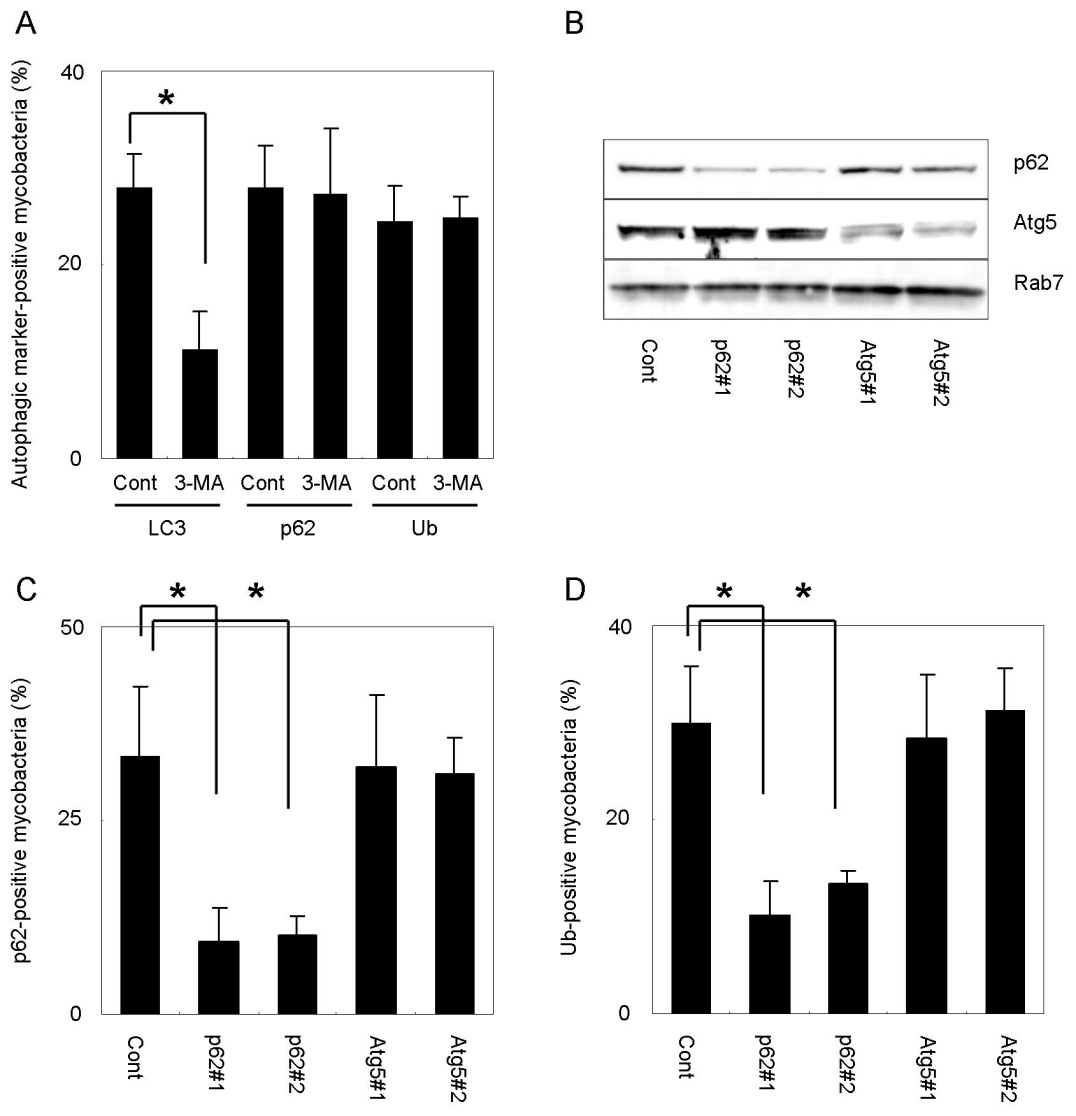
p62-dependent ubiquitination of mycobacteria in DC. (A) The proportion of LC3, p62 or ubiquitin recruitment to mycobacteria in DC treated with 3-MA. DC2.4 were infected with DsRed-expressing *M. tuberculosis* for 24 h with or without 3-MA and immunostained with anti-LC3, anti-p62 or anti-ubiquitin antibodies. Data represent the mean and SD of three or four independent experiments. *p < 0.05 (unpaired Student’s *t*-test). (B) Immunoblot analysis on the silencing effects of p62 and Atg5. DC2.4 cells were transfected with siRNA for p62 or Atg5 for 48 h and subjected to immunoblot analysis using indicated antibodies. (C, D) The proportion of p62 or ubiquitin recruitment to mycobacteria in DC. DC2.4 cells transfected with siRNA for p62 or Atg5 were infected with DsRed-expressing *M. tuberculosis* for 12 h and immunostained with anti-p62 (C) or anti-ubiquitin (D) antibody. Data represent the mean and SD of three independent experiments. *p < 0.05 (unpaired Student’s *t*-test).

To investigate the mechanism underlying autophagosome formation to *M. tuberculosis* in DC, we treated DC2.4 cells with 3-mehyladenine (3-MA), an autophagy inhibitor [35], and examined the localization of LC3, p62 and ubiquitin to infectied mycobacteria (Figure 6A). Treatment with 3-MA impaired LC3 recruitment to mycobacteria, but no significant changes were observed in the recruitment of p62 or ubiquitin. These results suggest that the classical autophagic pathway is not involved in the recruitment of p62 or ubiquitin to mycobacteria in DC. We next transfected DC2.4 cells with siRNA duplexes for p62 or Atg5 (Figure 6B) and examined the resulting effect on the localization of p62 or ubiquitin to mycobacteria in siRNA-transfected DC (Figure 6C, D). Both p62 and ubiquitin were recruited to mycobacteria in DC2.4 cells transfected with Atg5 siRNA, but the depletion of p62 decreased the ubiquitination of mycobacteria in these cells. Furthermore, p62 depletion, but not Atg5 depletion, decreased ubiquitinated mycobacteria in JAWSII cells (Figure S4). These results suggest that mycobacteria are ubiquitinated in a p62-dependent manner in DC.

### Atg5 functions in autolysosome biogenesis

**Figure 7:**
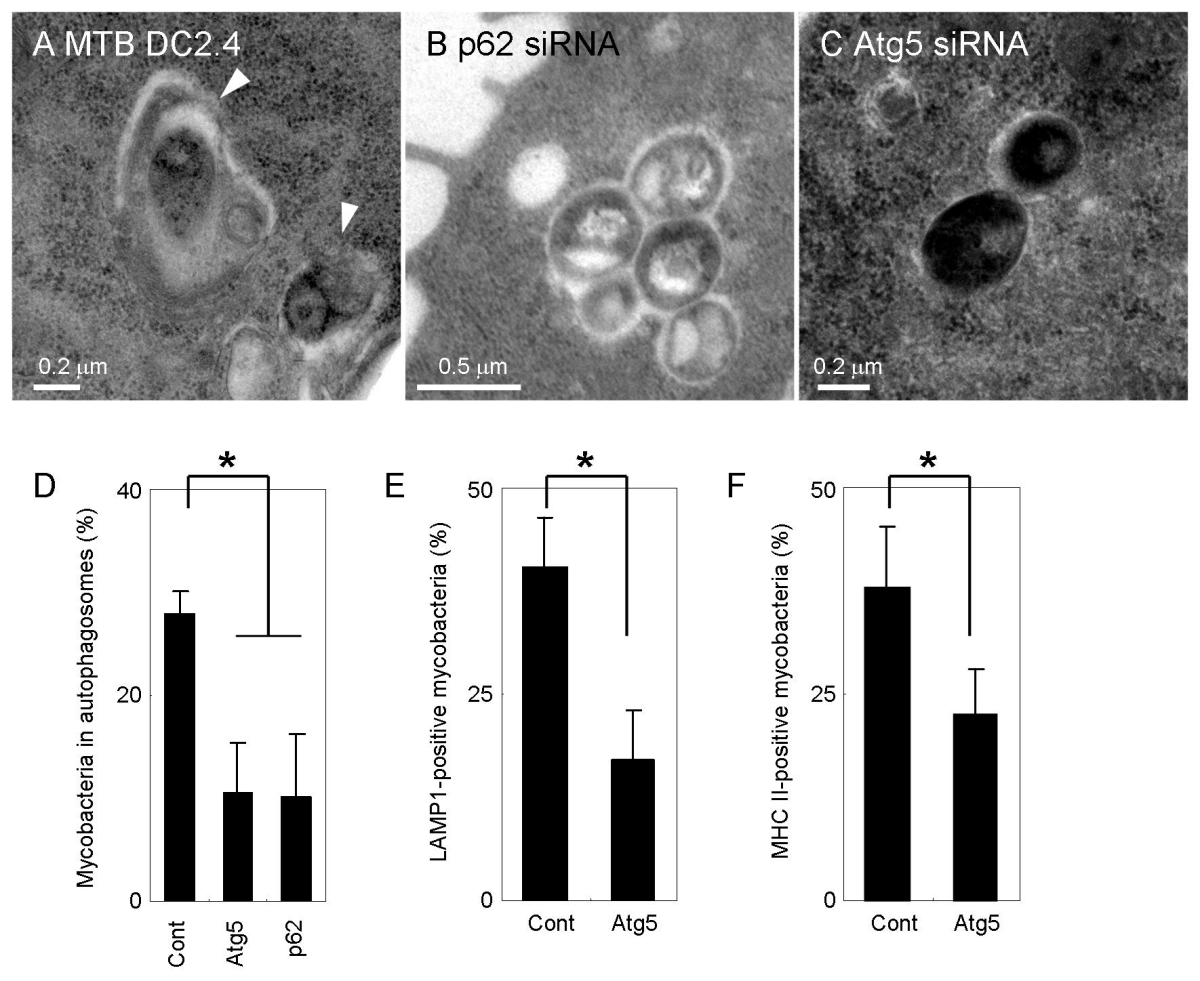
Atg5-dependent localization of LAMP1 and MHC class II to mycobacterial autophagosomes. (A-C) Thin-section electron micrograph of *M. tuberculosis* bacilli in p62- or Atg5-knockdown DC. DC2.4 cells transfected with siRNA for control (A), p62 (B) or Atg5 (C) were infected with *M. tuberculosis* for 24 h and observed by thin-section electron microscopy. Autophagosomes are indicated by arrowheads. (D) Proportion of mycobacteria in multi-membrane structures in DC2.4 transfected with siRNA for p62 or Atg5. (E, F) The proportion of LAMP1 (E) or MHC class II (F) localization to ubiquitin-positive mycobacteria is shown. JAWSII cells transfected with control or Atg5 siRNA for 24 h were infected with Alexa Fluor 405-labeled *M. tuberculosis* and immunostained with anti-LAMP1 and anti-ubiquitin antibodies or anti-MHC class II and anti-ubiquitin antibodies. Data represent the mean and SD of three independent experiments. *p < 0.05 (unpaired Student’s *t*-test).

To further investigate the function of Atg5 in autophagosome formation to mycobacteria in DC, we observed the ultrastructures of *M. tuberculosis*-infected DC2.4 transfected with p62 or Atg5 siRNA. Thin-section electron microscopy revealed that depletion of p62 or Atg5 inhibits autophagosome formation (Figure 7A-D), suggesting that Atg5 is also important for autophagosome formation to mycobacteria in DC.

Watson et al. demonstrated that Atg5 functions in autolysosome biogenesis to ubiquitinated *M. tuberculosis* in macrophages [36]. To determine the function of Atg5 in autophagosome formation to mycobacteria in DC, we examined the localization of LAMP1 or MHC II to ubiquitinated mycobacteria in JAWSII cells transfected with Atg5 siRNA (Figure 7E, F). Atg5 depletion decreased the proportion of LAMP1-positive or MHC II-positive ubiquitinated mycobacteria, suggesting that Atg5 functions in the fusion of lysosomes with mycobacterial autophagosomes in DC.

## Discussion

The induction of autophagy can eliminate *M. tuberculosis* in phagocytic cells [37,38], but the precise mechanism by which mycobacterial infection induces autophagy in phagocytic cells is not fully understood. Previous reports demonstrated that *M. tuberculosis* infection itself did not induce autophagy in infected macrophages [16,26,39]. However, other studies demonstrated that autophagosomes were formed in response to mycobacteria infection in macrophages [36]. Recently, it was demonstrated that *M. tuberculosis* infection induced autophagy but impaired the autophagic flux in human primary macrophages and DC [20,40]. In this study, we demonstrated that autophagosome markers localized to *M. tuberculosis* in BMDC but not in BMM (Figure 1). In DC cell lines, we also showed that autophagosomes are formed in response to *M. tuberculosis* infection (Figure 2), and found that lysosomal vesicles fuse with mycobacterial autophagosomes in DC (Figure 4).

Since autophagy is thought to be involved in antigen presentation via MHC II [22], it is assumed that autophagy promotes the presentation of mycobacterial antigens in DC. In this study, we demonstrated that *M. tuberculosis* infection induced selective autophagy in DC and that mycobacterial autophagosomes fuse with lysosomes followed by the recruitment of MHC II. These results suggest that the autophagosome formation in DC promotes degradation of infected mycobacteria and the resulting degradative peptides are then loaded onto MHC II, which are recruited to mycobacterial autophagosomes, leading the antigen presentation of mycobacterial peptides to CD4^+^ T lymphocytes via MHC II.

What is the initial event that triggers the induction of autophagosome formation to mycobacteria in phagocytic cells? The ubiquitination of bacteria or bacterial phagosomes is important for selective autophagy because ubiquitinated substrates are associated with the autophagy adaptor proteins which recruit LC3 and autophagosome membranes [41]. In *Mycobacterium marinum*, the ubiquitination of this bacterium is dependent upon the ESAT-6 and ESX-1 secretion system [42]. ESAT-6 of *M. marinum* disrupts the phagosomal membranes to assist bacilli escape from phagosomes to the cytosol [43-46]. *M. tuberculosis* can also damage the phagosome membrane and translocate from phagosomes to the cytosol in a mechanism that is dependent upon the ESAT-6 and ESX-1 secretion system [47,48]. In macrophages, the permeabilization of the phagosomal membrane signals the ubiquitination of *M. tuberculosis* [36,49,50]. Our results suggest that the initial step for ubiquitination of mycobacteria in DC is also triggered by the permeabilization of phagosomal membrane by ESAT-6 because BCG bacilli were not ubiquitinated in DC (data not shown). Recently, an E3 ligase was identified that interacts with NDP52 and is responsible for ubiquitination of *Salmonella* [51]. Manzanillo et al. demonstrated that a ubiquitin ligase, Parkin mediates the ubiquitination of intracellular bacteria in macrophages [50]. Results from the current study suggest the possibility that p62 mediates the recruitment of an E3 ligase for ubiquitination of *M. tuberculosis* bacilli or bacilli-containing phagosomes in DC.

Atg5 is involved in the elongation of isolation membrane in autophagosome formation [52]. We did not observe autophagosome membrane structures around infecting mycobacteria in DC by thin-section electron microscopy, however p62 and ubiquitin were recruited to mycobacteria in Atg5-knockdown DC (Figures 6 and 7). These results suggest that p62 and ubiquitin are recruited to infecting mycobacteria followed by the formation of autophagosome membrane depending on the function of Atg5. This hypothesis is consistent with the results that lysosomal markers do not localize to ubiqutinated mycobacteria in Atg5-knockdown DC (Figure 7). Atg16L is another autophagic-related protein involved in elongation of autophagic isolation membrane by forming an Atg5-Atg12-Atg16L complex [53]. We found that ubiquitin is recruited to *M. tuberculosis* but that LAMP1 does not localize to ubiquitinated mycobacteria in Atg16L-knockdown DC (Figure S5). These results suggest that ubiquitination of infected mycobacteria in DC is followed by formation of the autophagosome membrane and the autolysosome.

We found that autophagosomal markers did not localize to infected mycobacteria in macrophages, but did so in DC (Figure 1). These results imply that phagosomal membranes of DC are more susceptible to ESAT-6 and/or other secreted proteins from *M. tuberculosis* than those of macrophages. We have previously demonstrated that depletion of Coronin-1a leads the autophagosome formation to infecting *M. tuberculosis* in macrophages [26]. Coronin-1a associates with F-actin and localizes to mycobacterial phagosomes [54,55], suggesting that Coronin-1a supports the phagosomal membranes in macrophages but not in DC. However, proteomic analysis revealed that Coronin-1a localizes to mycobacterial phagosomes in both macrophages and DC [56], suggesting that other proteins localizing to mycobacterial phagosomes in macrophages but not in DC would contribute the autophagosome formation to infecting mycobacteria.

In conclusion, *M. tuberculosis* infection in DC induced autophagosome formation followed by the fusion with lysosomes and MHC II recruitment. p62 and Atg5 function in the initiation and progression of autophagosome formation to *M. tuberculosis*, respectively. Thus, p62 mediates the ubiquitination of *M. tuberculosis* and Atg5 is involved in the fusion of lysosomes with mycobacterial autophagosomes. These results imply that autophagosome formation in *M. tuberculosis*-infected DC contributes to activate CD4^+^ T lymphocytes via MHC II antigen presentation.

## Supporting Information

Figure S1 **Verification of co-localization between mycobacteria and autophagic proteins.** (i) Split the image into channels. (ii) Make the binary image for each channel. (iii) Merge the binary images to verify the co-localization.
(TIF)

Figure S2 **Quantification of band intensity for LC3-II.** The quantification of band intensity for LC3-II in Figure 2E was shown. The ratio of band intensity for LC3-II/tubulin at each condition to that of control DC2.4 cells is shown. Data represent the mean and SD of three independent experiments. *p < 0.05 (paired Student’s *t*-test).
(TIF)

Figure S3 **The proportion of LC3-positive (**A**), p62-positive (**B**) or ubiquitin-positive (**C**) *M. tuberculosis* in DC2.4 cells.** Data represent the mean and SD of three independent experiments.
(TIF)

Figure S4 **Ubiqutination of mycobacteria in JAWSII cells.** (A) Immunoblot analysis of JAWSII cells transfected with siRNA for autophagy-related genes. JAWSII cells transfected with siRNA for p62 or Atg5 genes for 48 h were subjected to immunoblot analysis using the indicated antibodies. (B) The proportion of ubiquitinated mycobacteria in JAWSII cells. JAWSII cells transfected with siRNA for p62 or Atg5 were infected with DsRed-expressing *M. tuberculosis* for 24 h and immunostained with anti-ubiquitin antibody. Data represent the mean and SD of three independent experiments. *p < 0.05 (unpaired Student’s *t*-test).
(TIF)

Figure S5 **Ubiquitination of mycobacteria in Atg16L-knockdown DC.** (A) Immunoblot analysis of DC2.4 cells transfected with siRNA for Atg16L. DC2.4 cells were transfected with Atg16L siRNA for 48 h and subjected to immunoblot analysis using anti-Atg16L antibody. (B) The proportion of ubiquitinated mycobacteria in Atg16L-knockdown DC. DC2.4 cells transfected with siRNA for Atg16 were infected with DsRed-expressing *M. tuberculosis* for 24 h and immunostained with anti-ubiquitin antibody. (C) The proportion of LAMP1 localization to ubiqutinated mycobacteria in Atg16L-knockdown DC. DC2.4 cells transfected with control or Atg16 siRNA for 24 h were infected with Alexa Fluor 405-labeled *M. tuberculosis* and immunostained with anti-LAMP1 and anti-ubiquitin antibodies. Data represent the mean and SD of three independent experiments. *p < 0.05 (unpaired Student’s *t*-test).
(TIF)
