# Supplementary figures and images for: Autophagy Adaptor Protein p62/SQSTM1 and Autophagy-Related Gene Atg5 Mediate Autophagosome Formation in Response to Mycobacterium tuberculosis Infection in Dendritic Cells
Source: PLoS One. 2013 Dec 23;8(12):e86017. doi: 10.1371/journal.pone.0086017 (PMC3871604; doi:10.1371/journal.pone.0086017)

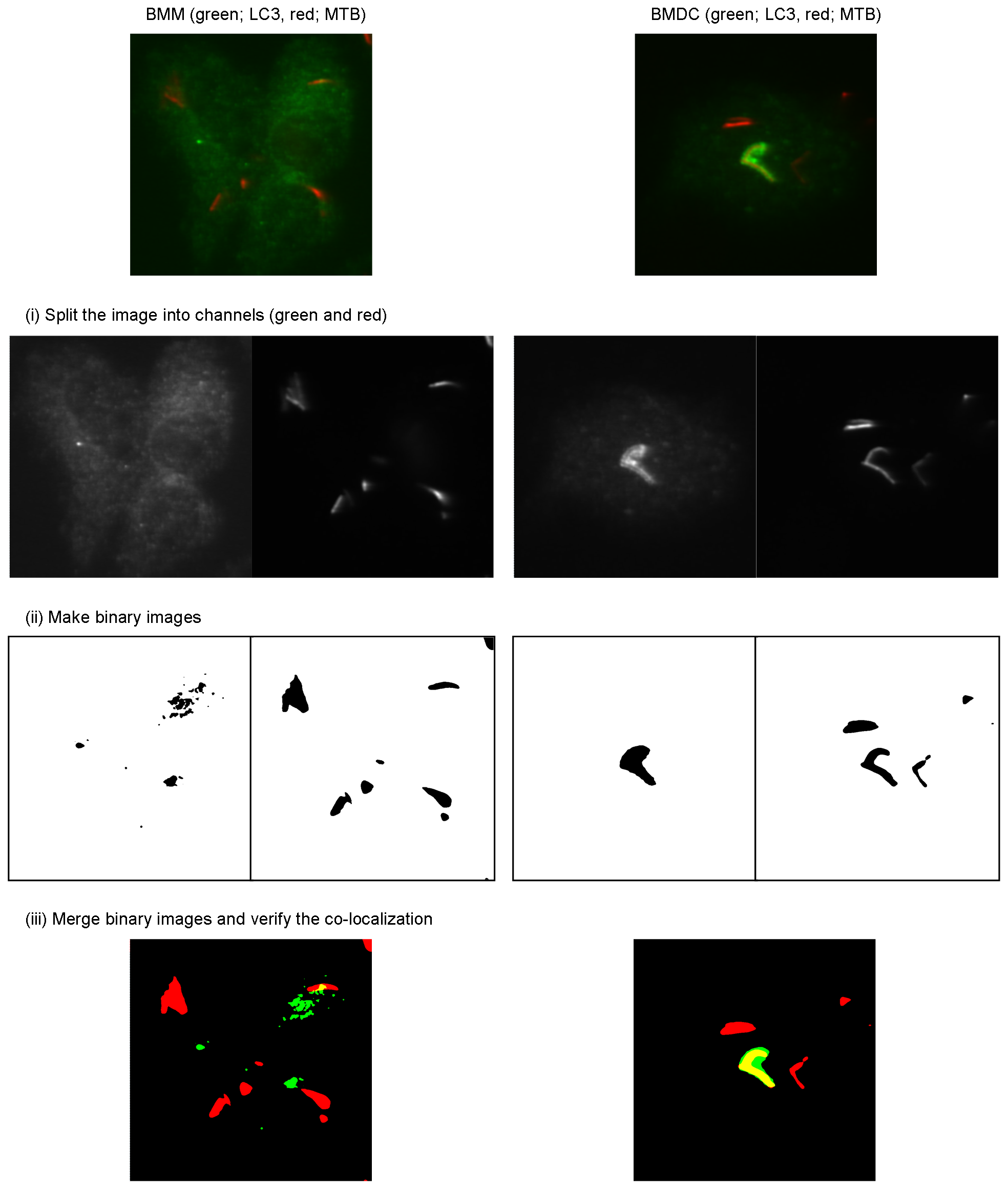

Supplement: Figure S1 — Verification of co-localization between mycobacteria and autophagic proteins. (i) Split the image into channels. (ii) Make the binary image for each channel. (iii) Merge the binary images to verify the co-localization. (TIF) [file pone.0086017.s001.tif]

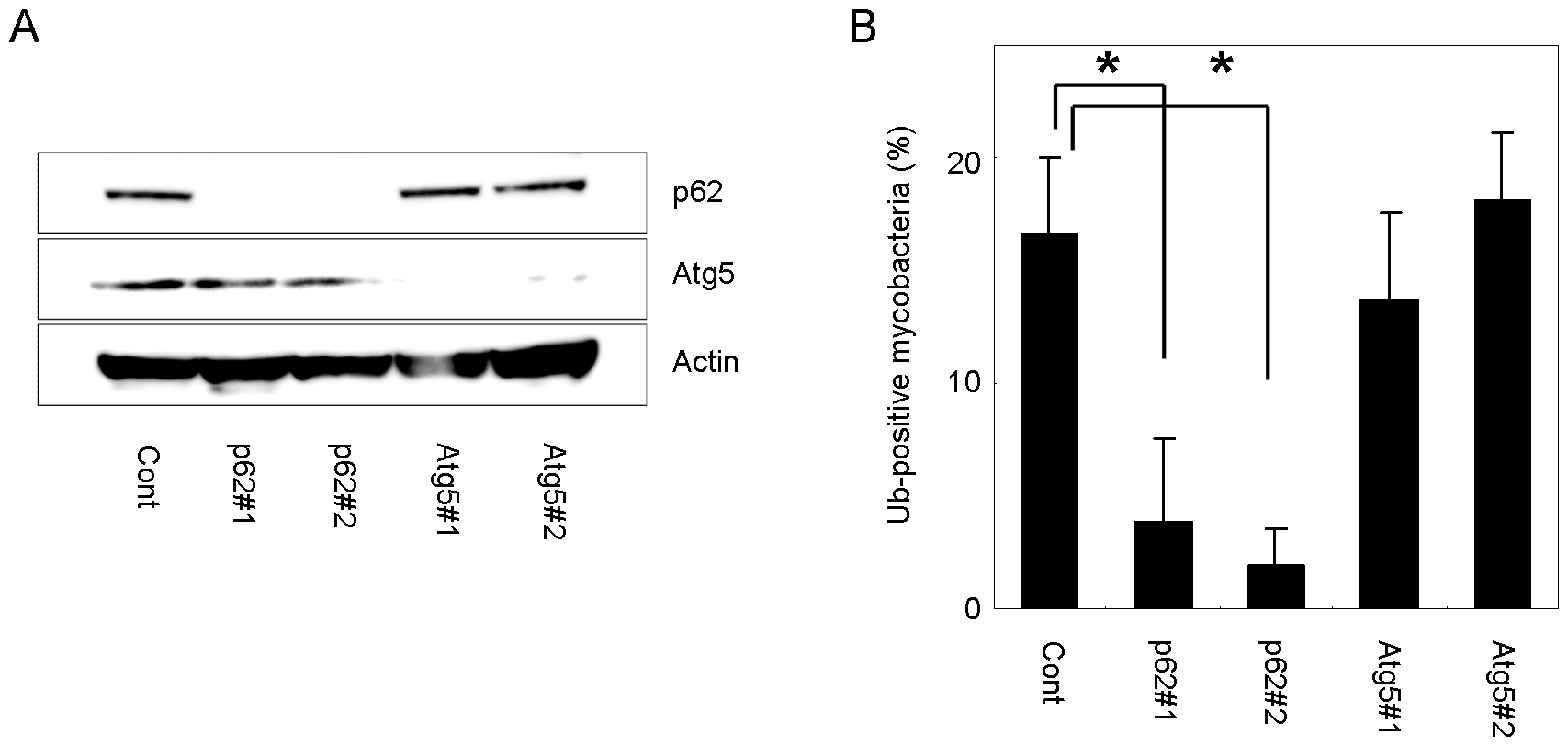

Supplement: Figure S4 — Ubiqutination of mycobacteria in JAWSII cells. (A) Immunoblot analysis of JAWSII cells transfected with siRNA for autophagy-related genes. JAWSII cells transfected with siRNA for p62 or Atg5 genes for 48 h were subjected to immunoblot analysis using the indicated antibodies. (B) The proportion of ubiquitinated mycobacteria in JAWSII cells. JAWSII cells transfected with siRNA for p62 or Atg5 were infected with DsRed-expressing M. tuberculosis for 24 h and immunostained with anti-ubiquitin antibody. Data represent the mean and SD of three independent experiments. *p < 0.05 (unpaired Student’s t-test). (TIF) [file pone.0086017.s004.tif]

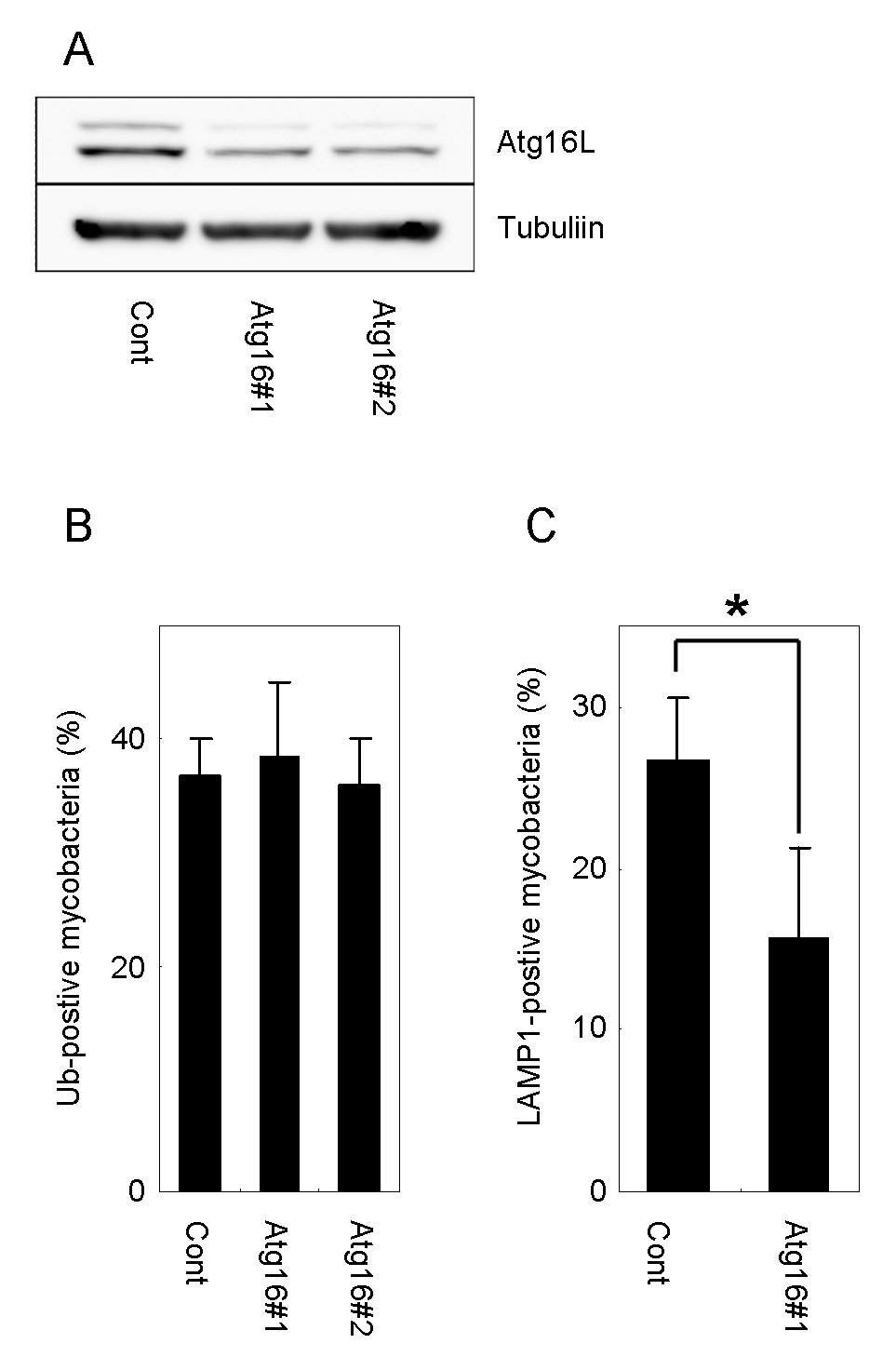

Supplement: Figure S5 — Ubiquitination of mycobacteria in Atg16L-knockdown DC. (A) Immunoblot analysis of DC2.4 cells transfected with siRNA for Atg16L. DC2.4 cells were transfected with Atg16L siRNA for 48 h and subjected to immunoblot analysis using anti-Atg16L antibody. (B) The proportion of ubiquitinated mycobacteria in Atg16L-knockdown DC. DC2.4 cells transfected with siRNA for Atg16 were infected with DsRed-expressing M. tuberculosis for 24 h and immunostained with anti-ubiquitin antibody. (C) The proportion of LAMP1 localization to ubiqutinated mycobacteria in Atg16L-knockdown DC. DC2.4 cells transfected with control or Atg16 siRNA for 24 h were infected with Alexa Fluor 405-labeled M. tuberculosis and immunostained with anti-LAMP1 and anti-ubiquitin antibodies. Data represent the mean and SD of three independent experiments. *p < 0.05 (unpaired Student’s t-test). (TIF) [file pone.0086017.s005.tif]
